# Circular RNA as an Additional Player in the Conflicts Between the Host and the Virus

**DOI:** 10.3389/fimmu.2021.602006

**Published:** 2021-05-28

**Authors:** Aditi Choudhary, Pratibha Madbhagat, M. Sreepadmanabh, Vipin Bhardwaj, Ajit Chande

**Affiliations:** Molecular Virology Laboratory, Department of Biological Sciences, Indian Institute of Science Education and Research (IISER) Bhopal, Bhopal, India

**Keywords:** circRNA, viral infection, innate immunity, host defense, host-virus interactions

## Abstract

Circular RNA (circRNA), a relatively new member of the non-coding RNA family, has spurred great interest among researchers following its discovery as a ubiquitous class within the RNA world. Rapid progress in circRNA biology has coincided with its identification in a plethora of diverse roles including regulation of gene expression and probable coding potential, as well as competing interactions with proteins and microRNAs in various pathological conditions. Emerging evidence suggests that circRNAs also function in viral infections. The deregulation of circRNAs during viral infection has prompted investigations into the possibilities of circRNA as a competing endogenous RNA (ceRNA) that modulates response to infection. Recently, viruses have been shown to encode circRNAs with proviral functions, providing a strong impetus for focused efforts to elucidate the networks coaxed by circRNAs during infection. This review elaborates on recent insights gained on the roles of circRNAs during virus infection and immunity.

## Introduction

The success of a pathogen is derived from its ability to subvert the host intracellular mechanisms effectively. Viruses, being obligatory parasites, are adept at subverting various host mechanisms for their benefit. Hijacking the host intracellular machinery by employing a set of virulence factors, viruses promote an environment within the host cell to produce millions of their copies and deplete the host resources. This sudden conflict must be solved, and the recurrence also has to be prevented from time to time. Hence, vertebrates have developed intricate antiviral signaling mechanisms with the help of which such intruders are kept in check. By augmenting the expression of antiviral factors comprising proteins and other endogenous non-coding RNAs (ncRNAs), the host strives to fight the incoming virus. Various viral and host strategies shape this battle. While central to this host-virus conflict has been proteinaceous effectors, recent evidence also suggests circRNAs as an arsenal employed by the host as well as the virus.

CircRNA is the class of RNA formed by a non-canonical splicing event, termed as back-splicing. Among other ncRNAs, the archaic perception of circRNA as transcriptional junk was a significant impediment to pioneering research on this topic (1). However, recent efforts have highlighted its omnipresence among vertebrate genomes, with a plethora of roles now being attributed- ranging from metabolic adaptation (2), regulating blood glucose homeostasis (3, 4), organellar circRNA dictating vital diseases (5, 6) to controlling lifespan (7) and aging (8). Here, we discuss the roles of circRNAs and their functions in viral infection outcomes.

## CircRNAs and the Cell-Intrinsic Antiviral Responses

Human cells have various proteins with antiviral functions that are augmented during the infection. These antiviral effectors target the viral life cycle and disrupt various virion components by sensing them as the virus tries to replicate. RNA viruses are detected by key RNA binding proteins such as retinoic acid-inducible gene I (RIG-I), melanoma differentiation-associated gene 5 (MDA5), laboratory of genetics and physiology 2 (LGP2), Protein Kinase R (PKR) and Toll-like receptor 3 (TLR3) (9), including among others. In contrast, DNA viruses are mainly sensed by cytosolic DNA sensors, which drive downstream signaling (10). Current understanding on the sensing of viral nucleic acids also underscores the potential immunogenic nature of the circRNAs. Concerning this, factors that could differentiate the self from non-self RNA circles have been identified, including the specific molecule-of-origin, the mode of biogenesis (11), circRNA-specific modifications (12), and cell type (13, 14). Further refinement of experimental approaches for studying immune responses to circRNAs would strengthen these observations in near future. Nevertheless, the differentiation of self from non-self is essential due to the fact that circRNAs are prevalent in eukaryotic cells as well as viral genomes and the possibilities of viral-mediated transport also is being envisaged. Innate immunity appears to be capable of detecting foreign circRNAs, but the molecular basis of self versus foreign identity awaits more research. Recent efforts have addressed this conundrum by identifying methylation as a switch that governs the immune response to circRNAs (12). Despite remarkable progress in deciphering the sensing of circRNA by the innate immune system, many questions remain unaddressed. For instance, the identity of additional factors and intracellular sensors governing the immunogenicity of circRNA in different cell types remains to be explored. Additionally, the ability of foreign circRNA or viral circRNA to differentially regulate innate immune response requires further investigations.

**Figure 1:**
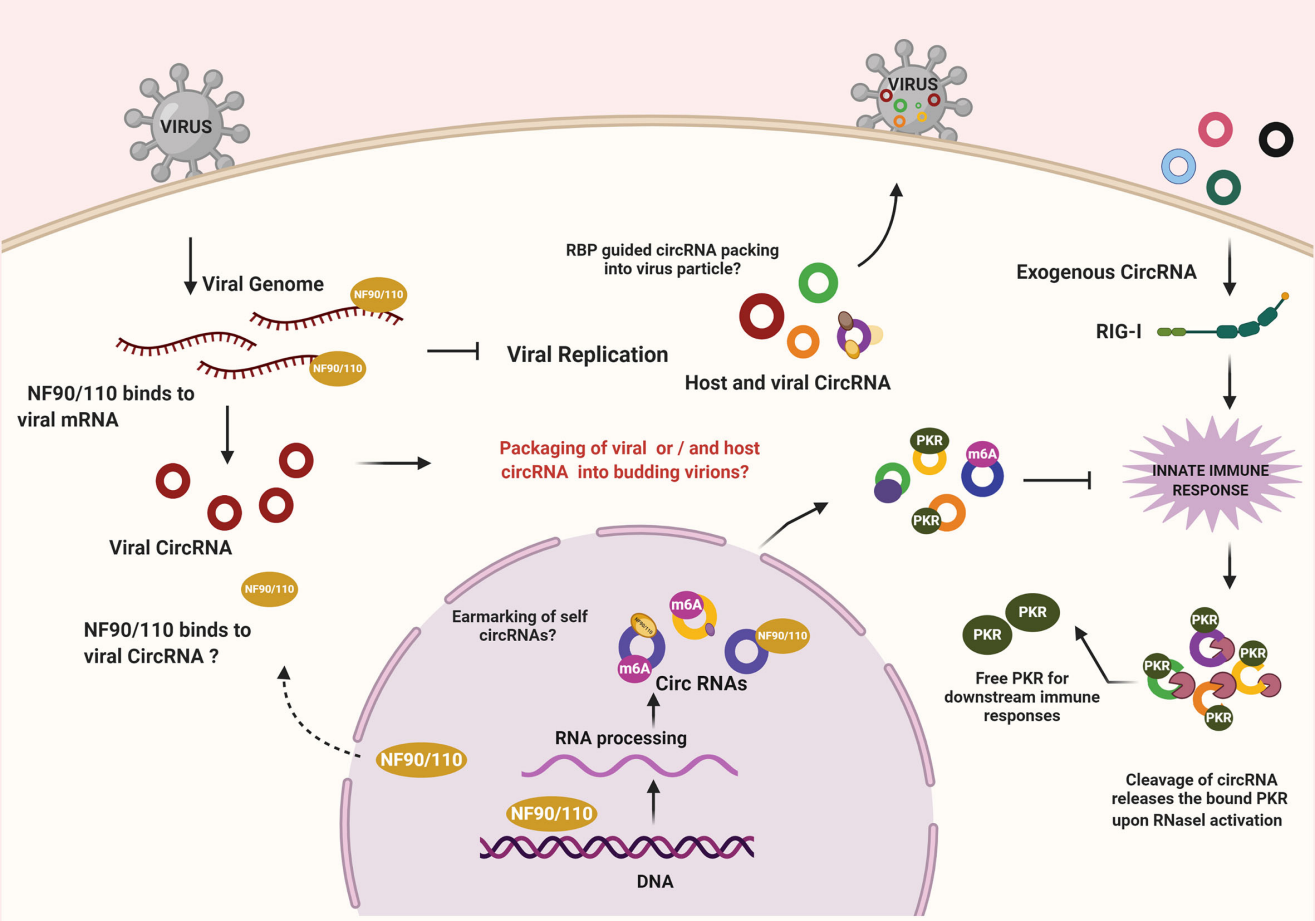
CircRNA mediated regulation of immune effectors during viral infections. NF90/NF110 promotes the biogenesis of circRNA within the nucleus. During infection, the export of NF90/NF110 to the cytoplasm leads to inhibition of viral replication. On the other hand, the exogenous circRNAs are sensed by RIG-I, leading to an innate immune response. Under normal conditions, the endogenous circRNAs escape recognition by immune sensing owing to the presence of mechanisms that confirms their self-origin. Cellular circRNAs can inhibit PKR, a key enzyme in antiviral signaling. RNase L-mediated degradation of circRNAs releases the locked PKR to promote antiviral state. The possibilities of RBP-driven packaging of circRNAs into viruses and the implications remains to be investigated.

Sensing of the viral infection activates a cascade of effector proteins within the host cell wherein the crosstalk and competition between host and viral factors begin (**Figure 1**). Recent studies have demonstrated the regulatory role of host circRNA in immunosurveillance. A study by Li and colleagues showed nuclear export of NF90/NF110 upon activation of the infection sensors like PKR to inhibit viral infection. Interestingly, NF90/NF110 in the nucleus promotes circRNA biogenesis and is often found in circRNP complex, however upon viral infection NF90/NF110 is released from circRNP complexes and binds to the viral RNAs - thereby inhibiting virus replication (15). It would be fascinating to identify other factors involved in pathogen sensing that intersect circRNA biogenesis or functions. An additional possibility is circRNAs acting as carriers for the host immune factors and is required to maintain the dormancy of immune mediators. A revealing study wherein yet another layer of the complex interplay between host circRNA and antiviral proteins was unearthed reported a locking mechanism that was enforced by circRNA to regulate immune responses. Characteristically, endogenous circRNA forms imperfect 16-26 bp RNA duplexes, which sponge PKR. However, upon viral infection, the endoribonuclease RNase-L depletes both viral and host RNAs, limiting the spread of the virus in the early stages. Because of the degradation of the associated circRNAs, PKR gets activated, which leads to the stimulation of innate antiviral immune responses *via* a downstream cascade (16). This suggests that by keeping the PKR dormant, circRNAs function as a “lock” by preventing spurious immune activation in undesirable conditions. It would be interesting to comprehend whether the activities of NF90/110 and RNase-L coordinates to elicit an effective response through PKR and, as yet unknown, other sensors in this context by modulating intracellular pools of circRNAs. Nonetheless, the ability of endogenous cia-cGAS (circular RNA antagonist for cGAS) to silence a critical DNA sensor cGAS in long-term hematopoietic stem cells (17) indicates the existence of more such mechanism(s) wherein circRNA abundance may determine the licensing of immune mediators. While these observations highlight immunomodulatory roles for circRNA, it also suggests the possibility that the defects in circRNA biogenesis factors or pathways may underlie autoimmune diseases.

## The Interplay of the Host and Viral-Derived CircRNAs

Parallel to the circRNA-mediated regulation of host factors, viruses also acquired various counteracting mechanisms that, as we now know, directly or indirectly involve circRNAs. Ultimately, this asserts a much complex interplay between the host and the viruses, which decides the overall infection outcome. A handful of exciting findings unveil the role of non-coding RNAs such as virus-derived small interfering RNAs (vsiRNAs) (18), PIWI-interacting RNAs (piRNAs) (18), and microRNAs (miRNAs) (19) as key players in such interactions. Due to its distinctive feature and unique way of acting as an intermediate in various biological processes, circRNA also has enthralled the scientific community to elucidate its role in the complex host-virus interplay.

For instance, Epstein-Barr Virus (a Herpesvirus) encodes circRPMS1 that was earlier shown to promote metastasis by escalating cell proliferation, cell invasion, and inhibiting apoptosis in EBV-positive Nasopharyngeal carcinoma (NPC) cells. It is also known to regulate gene expression by sponging miRNAs such as miR-203, miR-31, and miR-451 (20). Along similar lines, it was proposed that numerous miRNAs regulating both host and viral gene expression would be expressed during the viral latency phase of KSHV (Kaposi’s sarcoma-associated herpesvirus) infections in order to facilitate immune evasion (21–24). A few human-derived antiviral circRNA were reported to be activated upon viral infection for withstanding such viral immune exploitation. One such host-derived antiviral circRNA is hsa_circ_0001400, which gets induced upon KSHV infection and suppresses the expression of viral latent gene LANA and the lytic gene RTA. In response to such gene alterations, some viral circRNA expression is augmented in lytic infection vis-à-vis latent infection. Intriguingly enough, some KSHV-derived circRNA dampens the infected cell’s immunogenicity by inhibiting the viral gene expression itself (25). Such prevalence of circRNA-associated functions could provide insights for further investigation to understand viral antagonism mechanisms.

CircRNA was also found to regulate gene expression in the case of CHB (Chronic hepatitis B), promoting the pathogenesis of HBV (Hepatitis B virus) and associated liver disease. By establishing a bioinformatics pipeline for detecting circRNA associated with CHB and performing an in silico analysis of the circRNA-miRNA-mRNA axis, Zhou and colleagues have shown that the circRNA hsa_circ_0000650 promotes TGFβ2 expression by negatively impacting the miRNA, miR-6873-3p (26). Another finding by Chen et al. adds to the knowledge of circRNA as a defense mechanism. They studied the sponging of eIF4AIII (a crucial player in the Nonsense-Mediated Decay pathway) by circPSD3. During hepatitis C virus infection, expression of circPSD3 is enhanced, which leads to loss of available eIF4AIII and ultimately inhibits the NMD pathway (27). This inhibition may lead to the progressive accumulation of truncated proteins in the liver cells, which aids viral pathogenesis (28). However, pertinent questions remain unanswered. For one, being a host-derived circRNA, why would circPSD3 facilitate viral pathogenesis? Furthermore, acting as an RBP sponge for eIF4AIII its preferred mechanism of action?

To identify the role of circRNA in the host and viral interactions, researchers have developed competing endogenous RNA (ceRNA) networks (29) to explore circRNA mediated sponging that is equally compelling as other ncRNA. Here we discuss some of the studies done to understand the classical circRNA-miRNA-mRNA regulatory network that helped to explore host-virus interactions.

In Ebolavirus (EBOV) infection, both the immune system and the vascular system are hampered, which leads to severe hemorrhagic symptoms (30). To understand the disease progression, Wang et al. developed the complex ceRNA network revealing the interaction of circRNAchr19 and miR-30b-3p. This miRNA possesses potential binding sites in 3′-UTR of CLDN18, a tight junction gene (31, 32). The above interaction designates the putative function of circRNAchr19 to promote the expression of CLDN18 and evade sponging by miR-30b-3p. Similarly, in HTNV (Hantaan virus) infection, a study done by Lu et al. established the circ_0000479-miR-149-5p-RIG-I regulatory axis, which elucidates that sponging of miR-149-5p by circ_0000479 indirectly promotes RIG-I expression, thereby further inhibiting viral replication (33). Another finding *via* ceRNA networking showed that circRNA might serve as potential therapeutic targets in Middle East respiratory syndrome coronavirus (MERS-CoV) infection. An siRNA mediated knockdown of host-derived circRNAs, circFNDC3B and circCNOT1, significantly reduced viral load suggesting the pro-viral activity of circRNAs. Furthermore, circFNDC3B and circCNOT1 regulated target mRNA expression involved in ERK/MAPK pathway and RIG-I-mediated antiviral signaling (34). However, additional experimental evidence regarding such competitive interaction of circRNA with miRNA and target mRNA could reveal other potential gene regulatory effects in viral infections.

Our discussion until this point suggests that circRNAs are both liabilities as well as an asset for the host. Although the fate of virus-host interaction may be altered by circRNA, the probable answer would lie in the mechanism of action. It is very well conceivable that alternate mechanisms exist which presently remain unearthed.

## Future Perspectives

The discovery of circRNAs and associated functions has enabled a paradigm shift in the field of non-coding RNA biology, which has now extended to viral infections. Even considering just a handful of validated circRNAs, the interacting network seems intricately intertwined. Changes in the levels of circRNAs could have far-reaching ramifications on infection outcomes by modulating immune effectors. A wealth of intriguing early results on virally encoded circRNAs and those regulating immune responses provides a strong impetus for focused efforts in the future to elucidate their functions. Such analysis will require biochemical characterizations using relevant infection models to identify the interactions which are not only binary but include a network of potentially intertwined interactions coaxed by the circRNA.

In summary, an analysis of the myriad circRNA interactions in viral infections may represent a robust platform to uncover regulatory networks that protein-focused studies have overlooked. Development of circRNA-centric molecular assays and improvement in the functional assays could help unveil new roles of circRNA in the foreseeable future. Comprehensive insights into host-virus interactions obtained by such analyses can have exciting implications for developing an improved, enhanced, and effective therapeutic arsenal.

## Author Contributions

ACho and PM contributed equally. Conceptualization: ACha. All authors contributed to the article and approved the submitted version.

## Funding

ACha thanks DBT/Wellcome Trust India Alliance [grant number IA/I/18/2/504006] for the financial support. ACho, PM, and VB are supported by a fellowship from the MHRD, UGC, and CSIR, respectively.

## Conflict of Interest

The authors declare that the research was conducted in the absence of any commercial or financial relationships that could be construed as a potential conflict of interest.
